# A study on 3D imaging of maxillary sinus anatomical variations using CBCT

**DOI:** 10.6026/973206300213497

**Published:** 2025-10-31

**Authors:** Anuradha Chennupati, Mandarapu Satya Karthik, Harikrishna Modalavalasa, Bhargavi Katta, Prachi Krishna Kant Pandey, Supriya Shukla

**Affiliations:** 1 Department of Oral Medicine and Radiology, Government Dental College & Hospital, Vijayawada, India; 2 Department of Oral & Maxillofacial Surgery, Meghana Institute of Dental Sciences, Hyderabad, India; 3 Department of Prosthodontics, GITAM Dental College & Hospital, Visakhapatnam, India; 4 Department of Prosthodontics, Drs. S & NR SIDS, Gannavaram, Andhra Pradesh, India; 5 Prosthodontist & Implantologist, Mahavir Arogya Sansthan, Bihar, Patna, India; 6 Prosthodontist & Implantologist, Clove Dental Hospital, Noida, India

**Keywords:** Maxillary sinus, CBCT, anatomical variation, implant dentistry, oral surgery

## Abstract

The maxillary sinus, the largest paranasal sinus, shows considerable anatomical variations that influence oral and maxillofacial surgical procedures. In this retrospective CBCT study of 450 patients aged 18-75 years, sinus dimensions, septal patterns, floor morphology and proximity to posterior teeth were evaluated. The mean sinus volume was 14.7 ± 4.2 cm^3^, with bilateral asymmetry in 38.4%, septa present in 62.7% and root protrusions into the sinus observed in 41.3% of cases. The sinus floor-to-alveolar crest distance ranged from 1.2 mm at the first molar to 3.8 mm at the second premolar. These variations emphasize the necessity of CBCT imaging for precise surgical planning, particularly in posterior maxillary implant placement.

## Background:

The maxillary sinus, also known as the antrum of Highmore, represents the largest of the paranasal sinuses and plays a crucial role in various oral and maxillofacial surgical procedures [1]. Understanding the complex three-dimensional anatomy of the maxillary sinus is essential for oral surgeons, prosthodontists and radiologists involved in treatment planning for implant placement, sinus augmentation procedures and extraction of posterior maxillary teeth [2]. Traditional two-dimensional radiographic techniques, including panoramic and periapical radiographs, provide limited information regarding maxillary sinus anatomy and often fail to accurately depict anatomical variations that may significantly impact surgical outcomes [3]. The advent of cone beam computed tomography (CBCT) has revolutionized the assessment of maxillary sinus anatomy by providing high-resolution three-dimensional images with reduced radiation exposure compared to conventional CT [4]. Anatomical variations of the maxillary sinus include differences in size, shape, septal configurations, floor morphology and the relationship between the sinus floor and posterior tooth roots [5]. These variations can significantly influence surgical approach, risk assessment and treatment outcomes in procedures such as sinus floor elevation, implant placement in the posterior maxilla and endodontic treatment of posterior teeth [6]. The presence of antral septa, first described by Underwood in 1910, represents one of the most clinically significant anatomical variations [7]. These bony partitions can complicate sinus augmentation procedures and may increase the risk of sinus membrane perforation during lateral window approaches [8]. Similarly, variations in sinus floor morphology and the proximity of posterior tooth roots to the sinus cavity directly impact the feasibility and technique selection for implant placement [9]. Recent studies have reported considerable variation in the prevalence of maxillary sinus anatomical features across different populations, highlighting the importance of population-specific data for clinical decision-making [10]. Furthermore, age-related changes in sinus morphology, including pneumatization following tooth loss, emphasize the need for contemporary imaging assessment [11]. Therefore, it is of interest to describe the present study aimed to provide a comprehensive evaluation of maxillary sinus anatomical variations using high-resolution CBCT imaging in a diverse patient population, with a focus on determining their prevalence and clinical relevance in oral and maxillofacial surgical procedures.

## Materials and Methods:

## Study design and population:

This retrospective cross-sectional study analyzed CBCT scans obtained from patients who underwent imaging for various oral and maxillofacial indications between January 2021 and December 2023.

## Inclusion criteria:

[1] Patients aged 18-75 years

[2] High-quality CBCT scans with complete visualization of bilateral maxillary sinuses

[3] Field of view including the entire maxillofacial complex

[4] Absence of significant motion artifacts

## Exclusion criteria:

[1] History of maxillary sinus surgery or trauma

[2] Presence of sinonasal pathology

[3] Congenital craniofacial anomalies

[4] Orthodontic appliances causing significant artifacts

[5] Incomplete imaging data

## CBCT imaging protocol:

All CBCT scans were acquired using a Planmeca ProMax 3D Mid system (Planmeca Oy, Helsinki, Finland) with the following parameters:

[1] Tube voltage: 96 kVp

[2] Tube current: 12 mA

[3] Field of view: 16 x 13 cm

[4] Voxel size: 0.2 mm

[5] Exposure time: 13.9 seconds

Images were reconstructed and analyzed using Planmeca Romexis software version 6.2.

## Anatomical measurements and assessment:

Two calibrated oral and maxillofacial radiologists independently performed all measurements using standardized protocols. Inter-observer reliability was assessed using intraclass correlation coefficients (ICC).

## Primary measurements:

[1] Sinus volume: Calculated using semi-automatic segmentation tools

[2] Sinus dimensions: Anteroposterior, mediolateral and superoinferior measurements

[3] Septal analysis: Presence, location and morphology of antral septa

[4] Floor morphology: Classification based on Kwak *et al.* criteria [12]

[5] Root-sinus relationship: Distance measurements from root apices to sinus floor

## Secondary assessments:

[1] Bilateral asymmetry: Volume differences >20% between sides

[2] Septal classification: Complete vs. partial septa

[3] Pneumatization patterns: Zygomatic, alveolar and palatine recesses

[4] Accessory ostia: Presence and location of additional drainage pathways

## Statistical analysis:

Data analysis was performed using IBM SPSS Statistics version 28.0 (IBM Corp., Armonk, NY, USA). Descriptive statistics were calculated for all variables. Normal distribution was assessed using the Shapiro-Wilk test. Independent t-tests were used for comparisons between groups and chi-square tests were employed for categorical variables. Statistical significance was set at p < 0.05.

## Results:

Based on the study population demographics, a total of 450 CBCT scans from 450 patients (252 females, 198 males) were included in the final analysis. The mean age was 46.7 ± 14.2 years (range: 18-75 years). Age distribution showed 156 patients (34.7%) in the 18-35 age group, 189 patients (42.0%) in the 36-55 age group and 105 patients (23.3%) in the 56-75 age group (Table 1 - see PDF). Based on the Maxillary sinus volume and dimensions, the mean maxillary sinus volume was 14.7 ± 4.2 cm^3^ (range: 6.2-28.4 cm^3^). Significant bilateral asymmetry (>20% volume difference) was observed in 173 patients (38.4%). Males demonstrated significantly larger sinus volumes compared to females (16.2 ± 4.1 cm^3^ vs. 13.6 ± 3.8 cm^3^, p < 0.001). In Septal analysis, maxillary sinus septa were identified in 564 of 900 evaluated sinuses (62.7%). Complete septa extending from the sinus floor to the roof were observed in 208 sinuses (23.1%), while partial septa were found in 356 sinuses (39.6%). The most common location for septa was the middle third of the sinus (48.2%), followed by the posterior third (31.4%) and anterior third (20.4%).

Sinus floor morphology was classified into four types based on the relationship with posterior tooth roots:

[1] Type I (Flat floor): 186 sinuses (20.7%)

[2] Type II (Round floor): 342 sinuses (38.0%)

[3] Type III (Irregular floor): 258 sinuses (28.7%)

[4] Type IV (Deep recess): 114 sinuses (12.7%)

Root protrusions into the maxillary sinus were detected in 372 sinuses (41.3%), most commonly involving the mesiobuccal root of the first molar (28.4%) and the second premolar (19.7%). The variation in the sinus floor-to-alveolar crest distance across different posterior teeth is evident, with the first molar region showing the least available bone height (1.2 mm) and the third molar the greatest (4.2 mm), as detailed in Table 2 (see PDF). These findings emphasize the anatomical limitations for implant placement in the first molar region and the necessity of pre-surgical CBCT assessment.

In Pneumatization patterns, Accessory pneumatization was observed in various anatomical regions:

[1] Zygomatic recess: 278 sinuses (30.9%)

[2] Alveolar recess: 445 sinuses (49.4%)

[3] Palatine recess: 167 sinuses (18.6%)

[4] Infraorbital recess: 89 sinuses (9.9%)

Significant age-related increases in sinus volume were observed, with the 56-75 age group showing 13.0% larger volumes compared to the 18-35 age group (p < 0.01). Post-extraction pneumatization was evident in 156 cases (34.7%), predominantly in the first molar region.

## Male patients demonstrated:

[1] Larger mean sinus volume (16.2 vs. 13.6 cm^3^, p < 0.001)

[2] Greater prevalence of complete septa (26.8% vs. 20.2%, p < 0.05)

[3] More extensive pneumatization patterns (p < 0.01)

Inter-observer agreement for all measurements showed excellent reliability (ICC > 0.90 for continuous variables, κ > 0.85 for categorical variables).

## Discussion:

This comprehensive CBCT analysis of 450 patients provides valuable insights into maxillary sinus anatomical variations with significant clinical implications for oral and maxillofacial surgery and implant dentistry. The findings contribute to the growing body of evidence supporting the necessity of three-dimensional imaging for accurate assessment of maxillary sinus anatomy prior to surgical intervention. The observed prevalence of maxillary sinus septa (62.7%) aligns with recent meta-analyses reporting rates between 58-68% across different populations [13]. The presence of septa significantly impacts surgical planning for sinus augmentation procedures, as these structures can complicate membrane elevation and increase the risk of perforation during lateral window approaches [14]. Complete septa, observed in 23.1% of cases, may necessitate the creation of multiple windows or modification of surgical technique to ensure adequate access and visualization. The substantial bilateral asymmetry observed in 38.4% of cases underscores the importance of bilateral assessment even when unilateral treatment is planned. This finding has particular relevance for implant treatment planning, where contralateral sinus anatomy may influence the selection of implant length and surgical approach [15]. The close relationship between posterior tooth roots and the maxillary sinus floor, particularly in the first molar region (mean distance 1.2 mm), has significant implications for both endodontic and surgical procedures. Root protrusions into the sinus cavity, observed in 41.3% of cases, increase the risk of oroantral communication during extraction procedures and may complicate implant placement [16]. The variation in available bone height between different tooth positions (ranging from 1.2 mm at first molars to 4.2 mm at third molars) directly influences implant treatment planning and the need for sinus augmentation procedures. These findings support the recommendation for routine CBCT imaging prior to implant placement in the posterior maxilla [17]. The observed age-related increase in sinus volume (13.0% increases in the 56-75 age group) reflects the progressive pneumatization that occurs following tooth loss. This process, known as secondary pneumatization, results in decreased alveolar bone height and may complicate implant placement in older patients [18]. The high prevalence of post-extraction pneumatization (34.7%) emphasizes the importance of timely implant placement or ridge preservation procedures following tooth extraction in the posterior maxilla. The significantly larger sinus volumes observed in male patients (16.2 vs. 13.6 cm^3^) are consistent with general anatomical differences related to overall skull size [19]. However, the greater prevalence of complete septa in males (26.8% vs. 20.2%) represents a novel finding that warrants further investigation. These gender-related differences should be considered during treatment planning and risk assessment.

## Limitations and future directions:

This study has several limitations that should be acknowledged. The retrospective design and single-center nature may limit the generalizability of findings to other populations. Additionally, the exclusion of patients with sinonasal pathology may have underestimated the true prevalence of anatomical variations in the general population.

## Future research should focus on:

[1] Multi-center validation studies across diverse populations

[2] Longitudinal assessment of age-related anatomical changes

[3] Correlation of anatomical variations with surgical outcomes

[4] Development of AI-assisted automated measurement tools

[5] Investigation of genetic factors influencing sinus morphology

## Clinical recommendations:

Based on these findings, the following clinical recommendations are proposed:

[1] Mandatory CBCT imaging prior to all posterior maxillary implant procedures and sinus augmentation surgeries

[2] Bilateral assessment even for unilateral treatments due to high asymmetry prevalence

[3] Age-specific treatment planning considering progressive pneumatization in older patients

[4] Modified surgical techniques for cases with complete septa or complex anatomical configurations

[5] Patient counseling regarding anatomical variations and associated surgical risks

## Conclusion:

CBCT reveals significant variations in maxillary sinus anatomy, including septa, asymmetry and root proximity. These findings highlight the need for individualized, population-specific surgical planning. Routine CBCT use improves safety and outcomes in posterior maxillary implant dentistry.

## Author contributions:

## Study conception and design:

All authors contributed equally to the study design and methodology development.

## Data acquisition:

Primary data collection and CBCT analysis were performed by the oral and maxillofacial radiologists.

## Statistical analysis:

Statistical analysis was conducted collaboratively by all authors with primary responsibility shared between the oral surgeons and prosthodontists.

## Manuscript preparation:

Initial draft was prepared by the oral and maxillofacial radiologists with critical revisions provided by the oral surgeons and prosthodontists.

## Final approval:

All authors reviewed and approved the final manuscript for submission.

## Funding:

This research received no external funding. All imaging data were obtained as part of routine clinical care.

## Conflict of interest statement:

NIL

## Funding statement:

Nil
